# Can generative artificial intelligence enhance evidence-based and personalized medicine?

**DOI:** 10.1371/journal.pmed.1004931

**Published:** 2026-02-17

**Authors:** Arun J. Thirunavukarasu

**Affiliations:** International Centre for Eye Health, London School of Hygiene & Tropical Medicine, London, United Kingdom

## Abstract

In this Perspective, Arun Thirunavukarasu discusses a recent PLOS Medicine study comparing generative artificial intelligence (GAI) treatment recommendations with physicians', raising the question of whether GAI should be designed to follow treatment guidelines rigidly, provide individualized recommendations, or be somewhere in between.

## Teaser

Should clinical applications of generative artificial intelligence (GAI) be designed to follow treatment guidelines rigidly, provide individualized recommendations, or be somewhere in-between? A recent study in *PLOS Medicine* comparing GAI *versus* physician treatment recommendations highlights the importance of context- and patient-specific circumstances in decision-making.

The abilities of generative artificial intelligence (GAI) are continuing to expand, and clinicians and researchers are exploring and validating a growing number of clinical GAI applications [1]. The function of an application as well as the benchmark to which it is compared are active choices, and careful critical appraisal is essential to understand if a proposed solution is likely to be useful. In a recent *PLOS Medicine* study, Yang and colleagues [2] explored the potential impact of utilizing GAI to make treatment decisions for hepatocellular carcinoma (HCC) patients. Here, the authors assessed patients’ long-term survival in a retrospective registry-based study, comparing GAI decisions to clinician decisions as well as guideline recommendations [2].

In this retrospective study of 13,614 untreated HCC patients, three GAI models were tasked with providing treatment recommendations. Survival outcomes were used to estimate the impact of different treatment regimes on the HCC patients with disease stages ranging from Barcelona-Clinic Liver Cancer (BCLC) stage A to BCLC-C [3]. The concordance of GAI and clinician decisions ranged from 26.8% to 32.7%. Concordant decisions were associated with improved survival in early disease (BCLC-A) but worse survival in later-stage disease (BCLC-B and BCLC-C) [2]. Further analyses indicated that discordance in BCLC-A HCC was associated with lower blood albumin and platelet count, as well as higher international normalized ratio (INR); while in BCLC-C HCC, discordance was associated with higher albumin, and lower bilirubin and INR [2].

**Fig 1:**
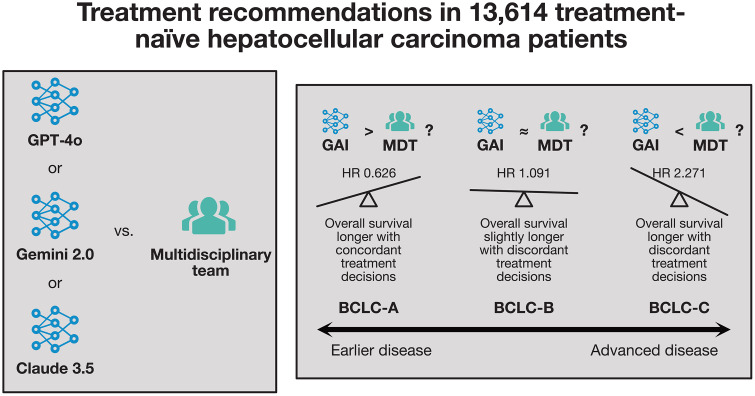
Comparing GAI and physician treatment recommendations for HCC patients [2]. Higher overall survival was observed where clinician and generative artificial intelligence (GAI) decisions were concordant in earlier hepatocellular carcinoma (HCC), and discordant in later disease [2]. This might suggest that GAI decisions could improve outcomes in earlier disease, but worsen outcomes in later disease. Ideally, prospective study is necessary to interrogate these conclusions. BCLC, Barcelona Clinic Liver Cancer classification schema; MDT, multidisciplinary team; HR, hazard ratio.

These results could mean that GAI decisions would improve outcomes in BCLC-A HCC patients, and worsen outcomes in BCLC-B and BCLC-C HCC (**Fig 1**). The discordance and survival results seem to be driven by the relative propensity of GAI and physicians to recommend curative therapy. Physicians appeared to place more emphasis on liver function, avoiding curative therapy in early HCC, where liver function was deemed limited and advocating curative therapy in later HCC when liver function was preserved (rather than adhering rigidly to treatment guidelines). In contrast, GAI was more likely to adhere to treatment guidelines—more intensive or local treatment of early disease and systemic or palliative therapy for late disease, with less influence of liver function. Of note, GAI did not have access to all of the data used by physicians, including raw imaging data which is critical for assessing the feasibility of surgical treatment. It is not clear whether multimodal GAI leveraging more data will behave in a similar fashion to the models tested in this study.

These inferences are tentative due to the inherent limitations of the observational study design. It is difficult to draw confident conclusions without prospective study, and randomization of HCC patients to GAI *versus* clinician-led treatment would be the best way to determine the true effect of GAI on overall survival—though currently ethically infeasible. Nevertheless, this study provides important insight into how GAI might fit into the clinical workflows, particularly as triadic care incorporating AI into the clinician-patient relationship is beginning to be discussed [1]. Guidelines are very helpful for promoting evidence-based care to patients, and can be used as a convenient ‘ground truth’ to assess the accuracy of GAI advice [4]. However, personalized care is also essential, accounting for the holistic clinical situation as well as individual circumstances and values. Do we want GAI to make an effort to mimic this idiosyncratic and nuanced process, or regurgitate guidelines with perfect recall?

There are situations where it is desirable for GAI to provide recommendations that are expected to be actioned, such as in efforts to broaden access to healthcare where access to clinicians is limited [5]. However, where clinicians are responsible for the care they provide, it may be more desirable for GAI to help improve efficiency by providing summaries of clinical information or mapping patients to relevant clinical guidelines. Physicians face a similar conundrum: Guidelines and clinical trials are designed to inform optimal decision-making, but treatments must be tailored to patients’ individual circumstances and preferences. For example, HCC experts cite various stratification tools and informative clinical studies to guide treatment, but also highlight the importance of multidisciplinary discussion and consideration of patient-specific factors [6]. The process of physician education and training should produce independent clinicians with an ability to weigh best available evidence with individual cases to provide the best healthcare possible [7].

A limiting factor for GAI developers is the information available to train and fine-tune models with. In medicine, the literature base skews towards ‘clinical research’ featuring groups sampled with the intention of drawing conclusions that generalize to broader populations. This includes randomized controlled trials, cohort studies, and case-control studies. In large part, this is because causal inference—such as determining the effect of a treatment—benefits from sufficient sample size and equilibration of factors that can bias results. GAI can draw on this evidence, as well as human synthesis projects such as clinical guidelines and systematic reviews, and may feasibly do better than any human at appraising the exponentially growing literature base [8]. However, the literature base alone does not govern best practice because individual circumstances matter: local resources, expertise, skills, values, and more [7]. For GAI to support individualized decision-making, it may benefit from accessing large corpora of idiosyncratic clinical narratives. This could require a change in priorities in clinicians’ publications. While clinical research and literature synthesis currently garner the most attention and citations [9], it may become a priority to disseminate case reports, individual decision-making, and multidisciplinary team reasoning. This might manifest via conventional peer-reviewed routes, partnerships between industrial GAI developers and healthcare systems, or a more open system making information generally available. While the latter option is preferable for maximizing opportunities for all to benefit, dissemination must be balanced with the principles of patient consent and confidentiality.

It may not be likely that GAI, in its current form, becomes capable or trusted to take on the complex role of a physician [10]. Even applications as assistants or sense-checks of clinician decisions entail significant risks such as failure to challenge flawed premises leading to confident but nonsensical recommendations [11]. As GAI improves in handling imaging and other multimodal data, performance will depend heavily upon its designed function [12]. GAI is likely to work best when tasked with roles that play to its strengths: synthesizing large volumes of data, answering factual questions where the required information is available, and completing repetitive tasks at a rate beyond any human [1]. Therefore, choosing to prioritize application of algorithms, collection and summarization of disparate data, or interrogation of individual circumstances and values, seems most appropriate to ensure that applications perform a clear and understood role in care, and that these applications enhance rather than confuse thought processes.

Ultimately, the role GAI plays in healthcare is up to us. Its desired functionality is context-specific, and clinicians are well-placed to describe and decide which applications may improve the care provided to patients. Being explicit about what we want GAI to do is crucial: From this, development, validation, and implementation methods follow.

## Abbreviations

BCLC: Barcelona-Clinic Liver Cancer
GAI: generative artificial intelligence
HCC: hepatocellular carcinoma
INR: international normalized ratio
